# How Veterans With Post-Traumatic Stress Disorder and Comorbid Health Conditions Utilize eHealth to Manage Their Health Care Needs: A Mixed-Methods Analysis

**DOI:** 10.2196/jmir.5594

**Published:** 2016-10-26

**Authors:** Julia M Whealin, Emily C Jenchura, Ava C Wong, Donna M Zulman

**Affiliations:** ^1^ Clinical Informatics Service VA Pacific Island Health Care System Honolulu, HI United States; ^2^ Department of Psychiatry John A Burns School of Medicine University of Hawaii Honolulu, HI United States; ^3^ Department of Psychology Arizona State University Tempe, AZ United States; ^4^ Center for Innovation to Implementation (Ci2i) VA Palo Alto Health Care System Menlo Park, CA United States; ^5^ Division of General Medical Disciplines Stanford University School of Medicine Stanford, CA United States

**Keywords:** stress disorders, post-traumatic, telemedicine, electronic mail, social media, self-care, computer literacy

## Abstract

**Background:**

Mental health conditions are prevalent among US veterans and pose a number of self-management and health care navigation challenges. Post-Traumatic Stress Disorder (PTSD) with comorbid chronic medical conditions (CMCs) is especially common, in both returning Iraq or Afghanistan and earlier war-era veterans. Patient-facing electronic health (eHealth) technology may offer innovative strategies to support these individuals’ needs.

**Objective:**

This study was designed to identify the types of eHealth tools that veterans with PTSD and comorbid CMCs use, understand how they currently use eHealth technology to self-manage their unique health care needs, and identify new eHealth resources that veterans feel would empower them to better manage their health care.

**Methods:**

A total of 119 veterans with PTSD and at least one CMC who have used the electronic personal health record system of the US Department of Veterans Affairs (VA) responded to a mailed survey about their chronic conditions and preferences related to the use of technology. After the survey, 2 focus groups, stratified by sex, were conducted with a subgroup of patients to explore how veterans with PTSD and comorbid CMCs use eHealth technology to support their complex health care needs. Focus groups were transcribed verbatim and analyzed using standard content analysis methods for coding textual data, guided by the “Fit between Individual, Task, and Technology” framework.

**Results:**

Survey respondents had a mean age of 64.0 (SD 12.0) years, 85.1% (97/114) were male, 72.4% (84/116) were white, and 63.1% (70/111) had an annual household income of < US $50,000. Mean score on a measure of eHealth literacy was 27.7 (SD 9.8). Of the respondents, 44.6% (50/112) used health-related technology 1 to 3 times per month and 21.4% (24/112) used technology less than once per month. Veterans reported using technology most often to search for health information (78.9%, 90/114), communicate with providers (71.1%, 81/114), and track medications (64.9%, 74/114). Five major themes emerged that describe how eHealth technology influences veterans with PTSD and comorbid CMCs: (1) interactions with social support, (2) condition management, (3) access to and communication with providers, (4) information access, and (5) coordination of care.

**Conclusions:**

The “Fit between Individual, Task, and Technology” model provided a useful framework to examine the clinical tasks that arose for veterans and their resourceful adoption of eHealth tools. This study suggests that veterans who use the Web are eager to incorporate eHealth technology into their care and self-management activities. Findings illustrate a number of ways in which the VA and eHealth technology developers can refine existing applications, develop new resources, and better promote tools that address challenges experienced by veterans with PTSD and comorbid CMCs.

## Introduction

Foremost among the challenges facing the US Department of Veterans Affairs (VA) is providing care for the more than 40% of VA patients who are suffering from mental health disorders [1]. The most commonly diagnosed mental health disorder at VA facilities is Post-Traumatic Stress Disorder (PTSD), occurring in 13% to 21% of veterans [1-5]. The symptoms associated with PTSD—intrusive memories, flashbacks (vivid recollections of the event), avoidance of stimuli associated with a trauma, negative mood, difficulty concentrating, and hyperarousal [6]—can substantially and negatively impact veterans’ abilities to cope with stress, function socially, and maintain employment [7-10]. Untreated PTSD is also associated with high rates of domestic violence, homelessness [11], and suicide attempts [12-14]. Not surprisingly, PTSD symptoms can impair veterans’ abilities to manage their health care needs [15].

To further complicate matters, PTSD often co-occurs with a number of chronic medical conditions (CMCs) [15,16]. Veterans with PTSD have high rates of circulatory, digestive, musculoskeletal, endocrine-nutritional, respiratory, infectious, and nervous system diseases [17-21]. In a national study of Iraq and Afghanistan veterans, 20% of men and 32% of women with PTSD had 10 or more diagnosed comorbid medical disorders [22]. These veterans can face especially complex self-management and health care navigation challenges associated with symptom control, treatment regimen adherence [23], and engagement in multiple medical services [24].

The potential for electronic health (eHealth) resources to better promote wellness in veterans with PTSD and comorbid CMCs depends on understanding patients’ health needs and preferences related to technology [25,26]. Among veterans in general, recent research suggests that about 70% of them access the Web [27], and of those veterans, many also use eHealth technology [28,29]. For example, veterans have been reported to access Web-based medical information [29], communicate with providers over secure messaging [30], access personal health records (PHRs) [31], and use Web-based and mobile apps to manage their symptoms [32]. Additionally, VA patients have been found to use social media to search for others with similar health problems at about the same rate as nonveterans [28].

To date, however, very little research has examined eHealth use among veterans with PTSD. One study that surveyed veterans who attended a VA PTSD outpatient clinic ([32]; N=188) found that 76% reported having access to a mobile phone or tablet device. Among this group, 85% expressed interest in accessing eHealth apps and 28% had accessed such an app. A study of 600 Iraq/Afghanistan era veterans [33] found that, compared with their peers, those who screened positive for PTSD were less willing to use various eHealth modalities, including online computer-based programs (50.6% vs 30.9%), text messaging (35.6% vs 24.3%), clinical telehealth (ie, the use of electronic media to facilitate real-time health care in the home; 52.7% vs 25.4%), and social networking sites (52.5% vs 34.8%). To better understand the needs and challenges facing veterans, Aponte and colleagues [29] recommended conducting follow-up studies on special populations of veterans, such as those suffering from PTSD and comorbid CMCs.

Given the multiple challenges that veterans with PTSD and comorbid CMCs face, it is vital to understand the ways in which the needs of these veterans interface with the types of eHealth resources available, as well as the health-related tasks veterans prefer and desire. The goals of this investigation were threefold: (1) to identify the types of eHealth tools used by veterans with PTSD and comorbid CMCs who use the Internet, (2) to understand how they currently utilize eHealth technology, and (3) to identify new eHealth resources that veterans feel would empower them to better manage their health care.

## Methods

### Study Design and Participants

We report on secondary data analyses from a sequential, mixed methods study comprising a survey followed by focus groups [34]. In 2012, a survey was mailed to a random sample of 1500 patients who receive care at a VA facility in northern California. Because the goal was to study individuals who use technology for health-related purposes, inclusion criteria for the mailing consisted of all patients who were registered users of the VA’s electronic PHR system. Of patients recruited, 479 responded to the survey (response rate of 31.93%, 479/1500). Of these, 119 patients reported having PTSD and at least one CMC and serve as the sample for the analyses reported here. Study procedures were approved by the Stanford University Institutional Review Board.

### Survey Design and Administration

For descriptive purposes, the survey asked patients to self-report their current health conditions using a list of 29 conditions that included “Post-Traumatic Stress Disorder.” Respondents could also write in additional conditions [34]. Because no validated and reliable item was available to assess comfort with technology when we developed this study, we adapted items from previous assessments of veterans [35] and nonveterans [36]. Respondents were asked: “Please describe how comfortable you are using the following types of technology,” with response options including “no experience” and a 5-point scale ranging from “very uncomfortable” to “very comfortable.” Participants then indicated whether or not they had experience using any of these technology modalities to help them manage their health care, the frequency of such use, and for what activities (eg, search for health information, buy medications or medical supplies, and communicate with providers).

The 8-item eHEALS (eHealth Literacy Scale) [37] was used to measure eHealth literacy, including respondents’ perceived knowledge, comfort, and skill at finding, evaluating, and applying electronic health information to health problems. Items are measured on a 5-point Likert-type scale ranging from 1 (strongly disagree) to 5 (strongly agree). The summed scores range from 8 to 40 [38]. This measure has been found to consistently capture the concept of eHealth literacy (coefficient alpha = .88; [39]). The scale correlates with consumer comfort and skill in using information technology. Principal components analysis produced a single factor solution (56% of variance).

### Focus Group Procedures and Content

Among the 119 individuals with PTSD and at least one CMC who completed the screening survey, 35 met eligibility criteria (29.4%, 35/119) for the focus groups (≥3 chronic conditions and experience using technology to help them care for their health or manage their health care, and having received care at the VA facility). Using purposive sampling, we constructed 2 focus groups of patients with PTSD, stratified by sex to enable a forum for discussing potentially sensitive topics (eg, military sexual assault). Sampling considerations included patients’ experiences with PTSD and multimorbidity, their experience with technology, and their availability to participate in a scheduled group. Of the 35 eligible patients with PTSD, 10 (29%, 10/35) participated in one of the focus groups.

The 2 sex-stratified groups consisted of 7 men and 3 women. Written informed consent was obtained from each focus group participant, and each received a US $50 gift card for participation. The focus groups were moderated by a trained research specialist using a semistructured guide [40]. The “Fit between Individual, Task, and Technology” (FITT) framework was used to guide the focus group content. The FITT framework is based on the idea that eHealth adoption depends on the match between the attributes of the user, the attributes of the technology, and the attributes of the clinical tasks and processes that a user needs to complete [41]. Thus, the framework was used to (1) examine the tasks that arise for veterans as a result of having to manage PTSD and comorbid CMCs, (2) determine current eHealth resources that aid patients with PTSD and comorbid CMCs in meeting these challenges, and (3) identify how eHealth resources could be further developed to better serve this population.

### Data Analysis

Descriptive summary statistics were computed for demographic and technology variables. Focus groups were transcribed verbatim and analyzed using standard content analysis methods for coding textual data [42]. As described elsewhere [40], transcript coding, supported by ATLAS.ti software (ATLAS.ti Scientific Software Development GmbH), was conducted as part of a larger project with guidance from qualitative research experts. Two research specialists then read through the documents to gain a sense of the data as a whole and then separately coded written responses for the groups with PTSD and comorbid CMCs to identify unique technology practices and needs.

## Results

### Participant Characteristics

Table 1 presents characteristics of survey respondents and focus group participants, facilitating qualitative comparison of characteristics such as age, sex, race/ethnicity, education, income, and chronic condition number. Survey respondents had a mean age of 64.0 (SD 12.0) years, 85.1% (97/114) were male, 72.4% (84/116) were white, and 63.1% (70/111) had an annual household income of < US $50,000. Focus group participants (n=10) had a mean age of 57.4 (SD 3.8) years, 70% (7/10) were male, 70% (7/10) were white, and 60% (6/10) had an annual household income of < US $50,000. Additionally, the large majority of the survey sample (92.4%, 110/119) and all focus group participants had 3 or more CMCs (Table 1). As shown in Table 2, the most common CMCs were chronic pain, high blood pressure, and arthritis or rheumatism, experienced by 57.1% (68/119), 49.6% (59/119), and 44.5% (53/119) of the entire sample, respectively, with comparable rates among focus group participants.

**Table 1:** Survey respondent characteristics.

Characteristics		Survey respondents without PTSD^a^ (n=347)^b^		Survey respondents with PTSD (n=119)^c^		Focus group participants (n=10)	
		Mean (SD)	n (%)	Mean (SD)	n (%)	Mean (SD)	n (%)
Age, years (N_SR_^d^=114, N_NoPTSD_^e^=343)		66.0 (11.3)		64.0 (12.0)		57.4 (3.8)	
Female (N_SR_=114, N_NoPTSD_=342)			23 (6.7)		17 (14.9)		3 (30.0)
**Race^f^ (N_SR_=116, N_NoPTSD_=316)**							
	White, non-Hispanic		289 (91.5)		84 (72.4)		7 (70.0)
	Black, non-Hispanic		14 (4.4)		11 (9.6)		0 (0.0)
	Hispanic (N_SR_=115, N_NoPTSD_=341)		21 (6.2)		12 (10.5)		2 (20.0)
	Other, non-Hispanic		28 (8.9)		9 (7.9)		2 (20.0)
**Employment^f^ (N_SR_=118, N_NoPTSD_=343)**							
	Full-time		58 (16.9)		12 (10.2)		—
	Part-time		37 (10.8)		6 (5.1)		—
	Retired		207 (60.4)		54 (45.7)		7 (70.0)
	Disabled		60 (17.5)		56 (47.5)		6 (60.0)
	Unemployed		35 (10.2)		20 (16.9)		2 (20.0)
	Student		4 (1.2)		7 (5.9)		2 (20.0)
**Education (N_SR_=118, N_NoPTSD_=342)**							
	High school degree or less		39 (11.4)		12 (10.2)		—
	Some college		131 (38.3)		62 (52.5)		6 (60.0)
	College degree or more		172 (50.3)		44 (37.3)		4 (40.0)
**Annual household income, US $ (N_SR_=111, N_NoPTSD_=320)**							
	<$50,000		193 (60.3)		70 (63.1)		6 (60.0)
	$50,001-$75,000		53 (16.6)		32 (28.8)		3 (30.0)
	>$75,001		74 (23.1)		9 (8.1)		1 (10.0)
**Total number of comorbid ** **conditions**		3.3 (1.9)		5.3 (2.3)		5.2 (1.3)	
	1-2		129 (37.2)		9 (7.6)		—
	≥ 3		212 (61.1)		110 (92.4)		10 (100)

^a^PTSD: Post-Traumatic Stress Disorder.

^b^Sample includes 347 individuals unless otherwise indicated.

^c^Sample includes 119 individuals unless otherwise indicated.

^d^N_SR_: number of survey respondents with PTSD.

^e^N_NoPTSD_: number of survey respondents without PTSD.

^f^For race and employment, participants could answer more than one.

**Table 2:** Chronic medical conditions reported by study participants.

Chronic medical condition^a^	Survey respondents without PTSD^b^ (n=347), frequency n (%)	Survey respondents with PTSD (n=119), frequency n (%)	Focus group participants (n=10), frequency n (%)
Chronic pain	130 (37.5)	68 (57.1)	6 (60.0)
High blood pressure	211 (60.8)	59 (49.6)	5 (50.0)
Arthritis or rheumatism	127 (36.6)	53 (44.5)	4 (40.0)
Diabetes	102 (29.4)	24 (20.2)	1 (10.0)
Depression	51 (14.7)	73 (61.3)	8 (80.0)
Lung or breathing problem	64 (18.4)	22 (18.5)	2 (20.0)
Prostate problems	64 (18.4)	15 (12.6)	1 (10.0)
Headaches or migraines	29 (8.4)	34 (28.6)	4 (40.0)
Cancer	34 (9.8)	18 (15.1)	2 (20.0)
Heart failure	30 (8.7)	7 (5.9)	2 (20.0)
Kidney problem	26 (7.5)	8 (6.7)	1 (10.0)
Chronic fatigue syndrome	10 (2.9)	10 (8.4)	1 (10.0)
Other	115 (33.1)	30 (25.2)	4 (40.0)

^a^Participants were able to circle more than 1 condition.

^b^PTSD: Post-Traumatic Stress Disorder.

Use of eHealth resources is presented in Table 3. Within the entire sample, 44.6% (50/112) of respondents used health-related technology 1 to 3 times per month and 21.4% (24/112) used technology less than once per month. eHealth technology was most commonly used to search for health information (78.9%, 90/114), communicate with providers (71.1%, 81/114), and track medications (64.9%, 74/114). The survey respondents and focus group participants were similar in that virtually all members of both groups had used and were comfortable using computers, the Web, and email. Similarly, both groups were less comfortable (and had less experience) using eHealth technology to visit support groups, make clinical telehealth calls, or participate in health-related mobile apps or games. Mean score on the eHEALS was 27.7 (SD 9.8) among survey respondents and 31.1 (SD 5.4) among focus group participants.

**Table 3:** Study participants with post-traumatic stress disorder and their comfort with and experience using technology for health-related purposes.

eHealth Technology Use		Survey respondents n (%)	Focus group participants n (%)
Health-related technology use (N_SR_^a^=116, N_FGP_^b^=10)		104 (90.0)	10 (100)
**Frequency of health-related technology use (N_SR_=100, N_FGP_=10)**			
	Daily	8 (8.0)	2 (20.0)
	1-5 times per week	18 (18.0)	1 (10.0)
	1-3 times per month	50 (50.0)	7 (70.0)
	Less than once per month	24 (24.0)	—
**eHealth activities (N_SR_=114^c^, N_FGP_=10)**			
	Searched for health information	90 (78.9)	10 (100)
	Communicated with provider	81 (71.1)	10 (100)
	Tracked medication list	74 (64.9)	9 (90.0)
	Tracked medical information	56 (49.1)	8 (80.0)
	Bought medications or supplies	40 (35.1)	5 (50.0)
	Made treatment decisions	49 (43.0)	9 (90.0)
	Visited online support group	15 (13.2)	3 (30.0)
	Used health-related mobile app (N_SR_=115)	19 (16.5)	4 (40.0)
	Participated in health-related competition or game	5 (4.4)	0 (0.0)
	Other	13 (11.4)	1 (10.0)
**Experience using technology (N_SR_=113, N_FGP_=9)^d^**			
	Computers (N_SR_=112)	112 (100)	9 (100)
	The Web	112 (99.1)	9 (100)
	Email (N_SR_=111)	110 (99.1)	9 (100)
	Text messaging (N_SR_=111, N_FGP_=8)	94 (84.7)	7 (87.5)
	Social media (N_SR_=111)	81 (73.0)	7 (78.0)
	Video calling	76 (67.3)	6 (67.0)
	Mobile apps	90 (79.6)	8 (89.0)

^a^N_SR_: number of survey respondents.

^b^N_FGP_: number of focus group participants.

^c^N_SR_=114 for eHealth activity unless otherwise specified.

^d^N_SR_=113 and N_FGP_=9 unless otherwise specified.

### Focus Group Themes

Five major themes describe how eHealth technology influences veterans with PTSD and comorbid CMCs, including their (1) interactions with social support, (2) condition management, (3) access to and communication with providers, (4) information access, and (5) coordination of care. Quotes from focus groups that represent each theme appear in an abbreviated version in Table 4. See Multimedia Appendix 1 for the complete list of quotes from the focus groups.

#### Interactions With Social Support

Four subthemes emerged that describe how technology influences veterans’ interactions with their social support.

##### Receiving Support

Veterans with PTSD and comorbid CMCs reported using technology to overcome difficulties connecting socially with others in person or via the telephone. In particular, texting and social media were seen as useful for connecting with others without the stressors of social interaction, such as becoming “too emotional.”

##### Providing Mutual Support

Several Veterans described using technology to facilitate mutual support within specific groups of veterans, for example women who attended a retreat together. Others stated that they were still seeking a means to connect with one another via social media. For example, a veteran who is attending a university was “trying to get our own place *(social media site)* to meet so that the veterans can stay together and kind of help each other with the school issues.” In other words, technology can allow veterans to connect with peers and maintain supportive relationships when face-to-face support is not feasible.

##### Obtaining Support to Cope With Symptoms or a Crisis

Veterans also reported using email and text messaging technology to give and receive support in times of crisis. Veterans indicated that they felt more comfortable using technology to connect with a peer or a professional who knew them, rather than someone they did not know, to help them cope with feelings of depression and suicidality. One veteran stated, “...when I got to my point where I was really at my lowest, you know, I called (*my therapist)* in the middle of the night and she arranged for someone to come pick me up...If I hadn’t had that, you know, I probably wouldn’t have gone to (the) emergency room or called 9-1-1 or called one of those crisis lines where you have to talk to some stranger.”

##### Deterring Social Support

Veterans also recognized that excessive use of technology, including social media, could promote avoidance of face-to-face socialization, which in turn sometimes fueled isolation. One veteran stated, “I think sometimes I feel safe on the computer or Facebook but...sometimes it doesn’t really get you out to meet people...Facebook is good but, sometimes, too much is not good.”

#### Condition Management

Three subthemes emerged that describe how technology is used to help veterans manage their medical conditions.

##### Using Web-Based Tools to Manage Symptoms

Veterans described using technology to access Web-based tools to help them cope with their PTSD and comorbid CMCs. For example, some veterans used mobile apps, such as games, to distract themselves when their anxiety elevated. Others used apps that were specifically designed to manage physical and psychological symptomatology, such as anxiety and high blood pressure. For example, veterans identified the VA “PTSD Coach” app [43] as particularly helpful. One veteran stated, “The (*smartphone*) is glued to my hand all the time and as soon as they got that PTSD app out...I loaded all my little pictures in there and my phone numbers and you can like send a text when you freak out and it will make a phone call (*to the crisis line*) for you.” Additionally, veterans used their digital devices to maintain a journal about the problems they encountered. One veteran opened a Web “blog” because he felt a website was a more secure location to write about his symptoms than in a book or on paper.

##### Providing a Sense of Safety and Security

Veterans stated that their mobile phones, in particular, have become a source of grounding and security and thus function to reduce anxiety when they are in public. One veteran stated, “You know, holding a...smartphone or whatever, you know, like I just have a rock in my pocket or something that will calm me down or focusing on something in the room to like kind of calm my anxiety.” Others indicated that following maps or directions was very stressful for them because of memory and concentration difficulties, and so navigation apps were helpful to reduce their anxiety about navigating on the road.

##### Signaling Reminders

Veterans described using computer programs, email, and mobile apps to keep track of a variety of health-related needs, including medical appointments and medication, as well as non–health-related tasks. Veterans identified the VA’s telephone system and Web portal (“My Health *e*Vet”) as being helpful for renewing and keeping track of medications. One veteran stated, “I utilize Google mail appointment reminders and I set them all and so they email me and they can message on my phone to tell me to order my medication, to take my medication, to make an appointment, to go to an appointment, and then tell (me) things I have to check off.”

#### Access to and Communication With Providers

Three subthemes emerged that describe how veterans use technology to improve access to and communicate with their providers.

##### Facilitating Accurate Reports of Pressing or Sensitive Issues

Many veterans reported that they were better able to convey the nature of their problems via digital technology than in person. Some felt that technology enabled them to share about important sensitive and/or stressful topics that were more relevant and accessible to them when they were outside the clinic. For example, veterans felt that being able to email their clinicians helped them share information that was embarrassing for them to talk about in person. Additionally, veterans felt that providing information from their home or natural day-to-day environment provided clinicians with a more accurate measure of their physical and mental health status. One veteran stated, “...when I was on active duty my psychiatrist and psychologist used email and it was good for them when I...could express how I felt at that time; for them to gauge my overall health status and not just what I say when I'm sitting in their chair. And they kept those as records to feed into my medical record so it helped them as much as it helped me.”

##### Promoting Timely Communication Between Veterans and Their Providers

Another theme focused on the convenience of engaging with providers via secure messaging. Veterans felt more connected to their providers and described that they and their providers could resolve problems more quickly using text messaging and email. One veteran stated, “Yeah, I think it’s faster for all of us, you know. It kind of frees up (providers’) time and they can answer (*my question*) when they can. And sometimes the conversation (*in office-based meetings*) goes a little bit longer because you don’t always think about what you are going to say and it kind of drags on more than what it needs to.”

##### Increasing Service Access for Disabled Veterans

Similarly, veterans stated that they either used or desired access to home telehealth care services when in-office health care sessions were not feasible. A common theme was the importance of health care sessions provided via technology for veterans disabled by mental and/or physical symptoms. One veteran stated, “For me like sometimes my migraines are really bad and a trigger for me and my migraines is driving so Skyping (*videoconferencing*) would be easier.” Other veterans indicated that technology could be helpful in situations when they are unable to attend health care sessions at the VA facility because of anxiety and hypervigilance secondary to agoraphobia or military sexual assault.

#### Information Access

Three subthemes emerged that described how veterans use technology to obtain health-related information.

##### Increasing Access to Trustworthy Health Information

Veterans reported that they often used technology to access “trustworthy” information from reliable websites that better enabled them to understand and manage their symptoms. A veteran stated, “I use the computer a lot. I use...other websites, the VA website. And so when the doctor tells me something then I can go and I can look and find resources or more information.” Another veteran reported using the VA Web portal as a source of information, stating “MyHealth *e*Vet...was a good program in order to find information and...be an advocate for my own health. There was a lot of information that I could use...and I could do it from home.”

##### Obtaining Information From Peers

Veterans described using the Web to access useful information from peers and/or veteran organizations. Veteran-specific blogs, social media sites, and organizations were particularly helpful. For example, one veteran stated, “...there’s a great blog for PTSD that covers PTSD individual unemployability, so I’m all over that. I don’t know who actually sponsors that blog but, every day there’s probably about 30 or 40 new questions or statements or something so that’s been really helpful.” Another stated, “Sometimes I just Google search a lot. I look for different woman veteran organizations, you know. Just to research and find stuff.”

##### Identifying Opportunities to Improve Means of Obtaining Health Information

Veterans expressed a need for consolidated health information presented in a way that did not lead to feeling overwhelmed by too much or, at times, conflicting information. Some reported difficulties in navigating information on the Web. For example, one veteran stated, “I went to the National Council for PTSD and got information. That led me off into a bunch of different directions so when you do your search on the Web you can either hit a good spot or a bunch of bad ones...Either way you’re getting a data overload.” To resolve this problem, one veteran suggested he would like to have “an app that would help show...some quick advice for things like PTSD for each of different conditions that you could go to that would help with these things.” Another veteran thought learning modules would be helpful, stating “log in and you could learn about all these different conditions...consolidated in one place and then it tracks what you studied and what you have learned and it gives you little reward points...making yourself feel good...”

#### Coordination of Care

Two subthemes emerged related to coordination of health care.

##### Improving Care Coordination Across Providers and Facilities

Instead of waiting for the health care system to create a formal link, veterans used technology to initiate treatment coordination between providers and, at times, across VA and non-VA facilities. One veteran was able to refill a prescription while on vacation. He stated “I ran into a situation where I was on vacation and I realized that I was running out of the medication...I sent my primary doctor an email saying, ‘Hey, I need this renewed.’” Another veteran stated, “So when I was starting to run out of medication, I went in and tried to look for my psychiatrist and I couldn’t. So I sent an email to my primary letting him know what was going on and what had happened. Well, my psychiatrist had forgotten to reissue my medication and they were able to communicate but she and I cannot communicate...but I was happy that it was acted on within a day or two. Good communication there...”

##### Identifying Opportunities for Improved Care Coordination

Veterans reported circumstances in which care coordination between facilities did not occur, leading to inconvenience. In one situation, for example, a veteran’s son was also enrolled in the VA for PTSD treatment but at a different VA facility than the veteran research participant. The veteran stated, “My son...had forgotten his medication. I took him down...to the VA here...they could not look up his...stuff in (his hometown VA), and be able to access it up here. So what I had to do is we had to sit there for two and a half hours and get him signed up in (my medical center).” Veterans wished for greater access to and coordination of records across VA facilities to expedite access to health care regardless of location and to afford providers knowledge of veteran health care activities across the VA.

**Table 4:** Focus group themes and sample statements describing eHealth technology’s influences for veterans with Post-Traumatic Stress Disorder and comorbid chronic medical conditions.

Themes and subthemes		Sample statements
**Interactions with social support**		
	Receiving support	“The Facebook effect is nice because sometimes you want to connect because you can't pick up the phone and you don’t want to see anybody in person but it’s that little tangible connection you can put something on and you get instant feedback. But I can be negative, too, if you don’t get any feedback.”
		“I use Skype all the time. My mother-in-law lives in (*another state)* so she sees the daughters on Skype. And I will Skype with someone.”
	Providing mutual support	“...my support system is mostly with (*women from a veterans group*). I text them because a lot of us don’t like to talk on the phone because sometimes you get too emotional. I just don’t feel like talking to people, but I text a lot of my woman vet and we support each other.”
		“I’m involved with the vet’s center pretty heavily in (*the city)*. I’m there once a week with a group and we all-all of us interact with each other like we’re doing right now, be it cell phone or a text or whatever away from the VA kind of, like what are you doing in terms of this, like in terms of mental health, in terms of losing weight. And I am also involved in a group through another added facet of the VA where we are all PTSD guys and we interact with them. So it’s all about networking with ourselves. Everybody in this room could be networking away from the VA. I don't know how healthy that is but it’s all via communication with our cell phones, primarily.”
	Obtaining support to cope with symptoms or a crisis	“...when I got to my point where I was really at my lowest, you know, I called (*my therapist*) in the middle of the night and she arranged for someone to come pick me up...if I hadn’t had that, you know, I probably wouldn’t have gone to emergency room or called 9-1-1 or called one of those crisis lines where you have to talk to some stranger.”
		“Because I have one psychologist in (*another city)* when my PTS was worse, I do not like to see anybody. I just-and then she send me an email, a poem, you know, why I should just don’t give up because I was suicidal. And she sent me that poem I just-you know, and I-just reading that poem kind of saved my life.”
	Deterring social support	“I think sometimes I feel safe on the computer or Facebook but...sometimes it doesn’t really get you out to meet people so that’s why I go on trying to find some social activity to do but I'm doing too much on the computer and I need to push myself out there. Facebook is good but, sometimes, too much is not good.”
**Condition management**		
	Using Web-based tools to manage symptoms	“The (*smartphone)* is glued to my hand all the time and as soon as they got that PTSD app out...I loaded all my little pictures in there and my phone numbers and you can like send a text when you freak out and it will make a phone call for you.”
		“I...use the PTSD app for my blood pressure because some of the imagery and the progressive relaxation helps me lower my blood pressure.”
	Providing a sense of safety and security	“...when I come to the VA I get really anxious and I see things that trigger me; men that get me angry and people in uniform. I just-I'm always holding my phone because having the Smartphone...Facebook and games, it has helped [my] mental health a lot...”
		“You know, holding a...smartphone or whatever, you know, like I just have a rock in my pocket or something that will calm me down or focusing on something in the room to like kind of calm my anxiety. Those are just some of the things that I use.”
	Signaling reminders	“...now that having a smartphone I have a task list. I put it on task so when I have an appointment or, you know, I put stuff on: tomorrow, don’t forget to go to MyHealth *e*Vet or reorder.”
		“The telephone system for renewing medications works great, you know at least in (*town)* where I go. And like you were saying, if your prescription is expired, they will automatically send a note to your doctor to request a new one, and that happens quickly. I mean it could take three or four days to get a prescription refilled if your prescription is expired and then getting another refill is probably maybe four days. So it’s really fast. So I don’t have any complaints...”
**Access to and communication with providers**		
	Facilitating accurate reports of pressing or sensitive issues	“...when I was on active duty my psychiatrist and psychologist used email and it was good for them when I would send them an email, I suppose, having like having a difficult time and I could express how I felt at that time; for them to gauge my overall health status and not just what I say when I'm sitting in their chair. And they kept those as records to feed into my medical record so it helped them as much as it helped me.”
		“They could make their visual check because there would be a lot of information on how you appear and they could probably learn more about you if they saw you in your home environment and not how you shower and put on clean clothes to come to the VA.”
	Promoting timely communication between veterans and their providers	“Yeah, I think (using email, secure messaging or texting is) faster for all of us, you know. It kind of frees up their time and they can answer it when they can. And sometimes the (*phone*) conversation goes a little bit longer because you don’t always think about what you are going to say and it kind of drags on more than what it needs to.”
		“About the email, the one thing that I really like is that the doctor has always got somebody waiting for him, so the nurses are the ones that were logging in to the email and doing the routing of the-and letting him know what’s going on, what the-and I really like that! That’s the sort of addresses the issue that you brought up.”
	Increasing service access for disabled veterans	“When I was on active duty I had the problem like you did. I didn’t want to go out of the house. I couldn’t get out of bed. I couldn’t take a shower. I couldn’t do anything. And they expect you, because I was on medical-waiting for my medical board, and they knew you couldn’t go to work but how can they expect you to get up and drive 45 miles for a doctor’s appointment when you can't even like feed yourself or take a shower?”
		“So when you have all those things, then it’s not making an hour out of your day for an appointment. You have to budget in well, it’s going to take me 15 or 20 minutes in the bathroom to clean myself up; it’s going to take me an hour, hour and a half before I can drive. Now we are talking, you know, two, three hours out of my day. So when I wake up in the morning do I really want to go? Do I really want to deal with it? I don’t want to deal with it. I have other stuff that’s more important. I'm just not going to, whereas, if it was just a Skype phone call then, I would be more likely to participate.”
**Information access**		
	Increasing access to trustworthy health information	“I use the computer a lot and the research-I use the Mayo Clinic and other websites, the VA website. And so when the doctor tells me something then I can go and I can look and find resources or more information.”
		MyHealth *e*Vet...was a good program in order to find information and...be an advocate for my own health. There was a lot of information that I could use...and I could do it from home.”
	Obtaining information from peers	“Because there’s a great blog for PTSD that covers PTSD individual unemployability, so I’m all over that. I don’t know who actually sponsors that blog but, every day there’s probably about 30 or 40 new questions or statements or something so that’s been really helpful.”
		“Sometimes I just Google search a lot. I look for different woman veteran organizations, you know. Just to research and find stuff.
	Opportunities to improve health information access	“I went to the National Council for PTSD and got information. That led me off into a bunch of different directions so when you do your search on the Internet (*Web*) you can either hit a good spot or a bunch of bad ones...Either way you’re getting a data overload.”
		“I use the Mayo Clinic and other websites, the VA website...The problem that I have is when I have multiple practitioners with different ideas about conditions as far as how to care for them or solve them or even their own interpretation of what the condition is—especially with PTSD.”
**Coordination of care**		
	Improving care coordination across providers and facilities	“I ran into a situation where I was on vacation and I realized that I was running out of the medication and it would take a certain amount of time once I got back to get it refilled. So I got on and I sent my primary doctor an email saying, ‘Hey, I need this renewed’ and I went in to look and see, no it hadn’t. So I had two days later come home, went in to see the pharmacist and she gave me a week’s worth of pills and she immediately put in a message to him, and that day he renewed the prescription. So it was really working well!”
		“So when I was starting to run out of medication, I went in and tried to look for my psychiatrist and I couldn’t. So I sent an email to my primary letting him know what was going on and what had happened. Well, my psychiatrist had forgotten to reissue my medication and *they* were able to communicate but she and I cannot communicate through the...but I was happy that it was acted on within a day or two. Good communication there...”
	Identifying opportunities for improved care coordination	“I had an experience. My son got out of the Marine Corps about three years ago. He...has PTSD in addition to some other issues. He takes an anti-anxiety medication and so he was up here over Christmas and had forgotten his medication. I took him down...to the VA here. It wasn’t that simple. I mean they could not look up his-even though he’s down in (*another*) county-he goes to the (*VA)* clinic down there, they could not look up his stuff in [*his hometown VA*], and be able to access it up here. So what I had to do is we had to sit there for two and a half hours and get him signed up in (my medical center)...it got him through it, but it was a pain...you know?”

## Discussion

### Principal Findings

This investigation used the FITT model [41] to identify how veterans used technology and eHealth resources to better manage their symptoms of PTSD and comorbid CMCs. The model provided a useful framework to examine the clinical tasks that arose for veterans and their resourceful adoption of technology and eHealth tools. Veterans with PTSD often suffer from severe mental health symptoms and multiple CMCs [22] that can substantially and negatively impact their ability to cope with stress, function socially, maintain employment [7-10], and manage their health care [15]. We found that veterans with PTSD who use the Web frequently used technology to creatively and effectively support their health care needs.

The veterans in this study reported moderate levels of perceived knowledge, comfort, and skill at finding, evaluating, and applying electronic health information. Most had searched for health information, communicated with a provider via email, and tracked their medication, but fewer had used online health-related support groups, games, and mobile apps. The average eHEALS (eHealth Literacy Scale) score was comparable to scores reported by similarly aged nonveteran samples, including 283 baby boomers and older adults who use the Web (mean age 67.5 years; mean eHEALS score 29.1, SD 5.8) [44] and 866 adults aged 50 years or older, who use the Web (mean age 62.8 years; mean eHEALS score 30.9, SD 6.0) [39]. The relatively high self-reported eHealth literacy reported in these samples may be due to their high education levels and experience using the Web and, in the case of our study, purposeful sampling of those who had exposure to the VA’s electronic patient portal system [44].

Our qualitative findings identified eHealth resources that empowered veterans to better manage their health care for their PTSD and comorbid CMCs. Findings suggest that health care systems should promote technology that addresses 5 themes: (1) interactions with social support, (2) condition management, (3) access to and communication with providers, (4) information access, and (5) coordination of care. Focus group themes aligned with our quantitative findings, which showed that the most common use for technology was to search for health information (consistent with the theme “information access”). Moreover, the second and third most common uses for technology were to communicate with providers and to track medications, respectively. These uses corroborated the focus group themes “access to and communication with providers” and “condition management.” As described below, qualitative findings went beyond quantitative results to more comprehensively define how veterans actually used technology to manage their health care needs and to prevent potentially injurious problems from occurring.

### Implications of Focus Group Results

Social support is a consistent correlate of positive outcomes in veterans with PTSD [45]. However, PTSD symptoms such as hypervigilance, negative mood, emotional numbing, and avoidance of reminders of the traumatic event make obtaining and maintaining social support difficult. In this study, one attribute of technology that emerged as a potent resource for veterans with PTSD and comorbid CMCs was its ability to facilitate social support. Veterans used email, texting, social media, and blog discussion sites to connect with others for mutual encouragement, informational advice, and tangible support regularly, as well as in times of crisis. Connecting with peers for encouragement helped veterans cope with difficult psychological symptoms that often cause trauma survivors to isolate from others. Our findings support limited empirical data suggesting that peer support may positively impact mental health symptoms [46-48].

Veterans who suffer from PTSD symptoms such as hypervigilance, negative mood, and flashbacks can experience high levels of stress when in public settings. As a result, veterans with PTSD and comorbid CMCs may avoid grocery stores, health care appointments, social gatherings, and other events [49,50]. In this study, mobile phones were found to be an important source of grounding and security that enabled veterans to better function in public settings. In addition to knowing that they could use their phone to access a support person, veterans used specific eHealth tools, such as the VA “PTSD Coach” mobile app [43], to cope with their difficult symptoms. Interestingly, only a minority of participants (16.5% (19/115) of survey respondents and 40.0% (4/10) of focus group members) in this study indicated that they had used a health-related mobile app. Other research on veterans with PTSD who own a mobile phone [32] has found that 28% have heard of or used specific apps related to PTSD (ie, “PTSD Coach”). However, 85% of veterans in that study who owned devices expressed interest in using mobile apps to address health related-issues. Thus, veterans who are not aware of such resources will likely benefit from learning about existing apps.

In addition to directly managing their symptoms, participants used electronic devices to increase communication with providers by mobile phone and by secure email messaging. Veterans felt that communication was more accurate and candid when delivered via digital modalities compared with face-to-face settings. Veterans also described feeling better able to report symptoms more accurately at the time they were occurring, which is consistent with some research on military populations [51,52]. Additionally, because veterans feel more comfortable in their home settings (compared with clinic settings), they also described feeling better able to provide sensitive information, such as thoughts of suicide, more candidly when using technology. Veterans with PTSD are at increased risk for suicide [12-14] and interpersonal violence [11-14], so tools that promote candid and timely communication will be essential to prevent potentially injurious outcomes from occurring.

Another modality embraced by the VA to increase access for veterans is clinical videoconferencing, which directly connects providers to veterans who are located in their homes or in another VA clinic [53,54]. Veterans in our investigation expressed interest in clinical videoconferencing sessions to address active symptoms. However, one symptom of PTSD is avoidance, or the tendency to withdraw and/or disengage from social settings and day-to-day life events [55-58]. It is important to caution that the exclusive use of home-based appointments may not be in the best interest of some veterans with PTSD. Care must be taken to ensure that virtual services do not prevent veterans from actively engaging in healthy life events.

Moreover, whereas research supports the role of technology in increasing social support and reducing isolation [59], veterans in this study recognized that excessive use of technology, including social media such as Facebook, can promote avoidance of face-to-face socialization among veterans with PTSD. We are not aware of research that has examined the effect of technology on social support in those with PTSD and comorbid CMCs, but our results support our clinical experience suggesting that technology can be used by those with PTSD and comorbid CMCs to promote isolation ([60, page 49]; [61]). Clinical intervention may be necessary to encourage veterans with PTSD and comorbid CMCs to approach technology in a manner conducive to recovery.

Veterans used technology to access health information, but some indicated that they have become overwhelmed by the amount of resources available and, at times, by conflicting information. Materials designed for veterans with PTSD and comorbid CMCs should take into consideration the mild to moderate cognitive impairment that can be associated with their symptoms. There additionally appeared to be a desire for a trusted service that distinguishes high-quality consumer information from biased or lower-quality materials. Veterans need information presented in a format and at an educational level that engages them [54], and they may benefit from guidance when sifting through seemingly disparate and/or conflicting materials.

Finally, veterans used technology to initiate treatment coordination between providers and, at times, across facilities. This care coordination was particularly important because study participants were often tasked with coordinating care for different health issues from multiple providers [40]. Veterans in this study also reported instances when technology was not available to promote care coordination between facilities. Thus, providing veterans with greater access to records across health care institutions via patient-facing apps and/or Web portals will better empower veterans to quickly access health resources regardless of their location, improving the VA records system and facilitating communication across systems of care.

### Limitations

The purpose of this research was to understand veterans’ experiences using eHealth technology to help manage their unique PTSD and comorbid CMC symptoms. Because our goal was to identify how veterans use eHealth, we purposefully selected from users of the VA's patient portal system (My Health *e*Vet) in order to restrict the sample to veterans who have had Web exposure, which limits the generalizability of the results. Results are also limited by our survey response rate of 31.93% (479/1500). Survey respondents may be predisposed to view eHealth positively. We attempted to minimize this bias by offering veterans remuneration for their efforts filling out the survey. Additionally, participants in this study were primarily Caucasian veterans selected from a relatively computer-literate region in northern California. Their perspectives may be different from veterans who do not have access to or prefer not to use the Web, reside in other regions, or are of minority status. Finally, focus group members consisted of a small subgroup of veterans whose attitudes may not represent all veterans with PTSD and comorbid CMCs who use the Web. However, focus group members were similar to other study participants in that virtually all members of both groups had used and were comfortable using computers, the Web, and email but had less experience using online health-related support groups, games, and mobile apps. Additionally, eHealth literacy scores of veterans in the survey sample and focus group subsample were similar to those found in other samples of similarly aged adults who use the Web [41].

### Recommendations and Summary

Our study suggests a number of opportunities to support veterans with PTSD and CMCs through eHealth technology. First, study participants expressed interest in increasing contact with peers through technology, for example, through social media groups for veterans in college or clinician-monitored chat rooms for group therapy patients to support one another between visits. Second, eHealth technology can provide symptom management support, for example, through mobile apps [62] or live Web-based classes. The VA continues to develop its mobile apps patient portal system, and individual staff members are in an ideal position to help disseminate effective tools [63,64]. Third, providers can capitalize on veterans’ desire to use eHealth to access help when they need it. In addition to mobile apps, modalities for development might include texting, email, and/or blog sites. Fourth, using digital technology, veterans can consolidate new life skills by completing “homework” assignments between therapy sessions. Timely feedback from remote clinicians can help maximize the relevance and effectiveness of such tools. Fifth, veterans expressed a need for consolidated health information. It is clear that veterans need educational materials presented at an appropriate level and in an engaging format [54]. To this end, the VA has adopted a patient-centered model of care with a focus on coordination of information technology [40,64] and is leading efforts to understand how technology may be adapted to meet individuals’ needs [25,64].

In summary, the results of this investigation help establish that veterans with PTSD and CMCs who use the Web are eager to incorporate eHealth technology into their care and self-management activities. Furthermore, study findings suggest opportunities to augment the potential power of eHealth as an adjunct to care, particularly with regard to preventive care. The themes that emerged from this investigation help characterize approaches the VA and eHealth technology developers can take to refine existing resources and develop new tools to better serve veterans with PTSD and CMCs. Future research should evaluate whether such patient-centered endeavors facilitate the appropriate use of health care services and improve clinical outcomes.

## Appendix

Multimedia Appendix 1 Focus group themes describing eHealth technology’s influences for veterans with Post-Traumatic Stress disorder and comorbid chronic medical conditions.

## Abbreviations

CMC: chronic medical condition
eHEALS: eHealth Literacy Scale
eHealth: electronic health
FITT: Fit between Individual, Task, and Technology
PHR: personal health record
PTSD: Post-Traumatic Stress Disorder
VA: US Department of Veterans Affairs
