# Supplementary material for: How Veterans With Post-Traumatic Stress Disorder and Comorbid Health Conditions Utilize eHealth to Manage Their Health Care Needs: A Mixed-Methods Analysis
Source: J Med Internet Res. 2016 Oct 26;18(10):e280. doi: 10.2196/jmir.5594 (PMC5103157; doi:10.2196/jmir.5594)
Supplement: Multimedia Appendix 1 [file jmir_v18i10e280_app1.pdf]

Multimedia Appendix 1

Focus group themes describing eHealth technology's influences for veterans with PTSD and comorbid CMCs

Interactions with Social Support
**Receiving Support**<br><br>o "The Facebook effect is nice because sometimes you want to connect because you can't pick up the phone and you don't want to see anybody in person but it's that little tangible connection you can put something on and you get instant feedback. But I can be negative, too, if you don't get any feedback"<br><br>o "Sometimes I feel safe on Facebook, just-I mean, I mostly get my support from not my family but-my family, I don't try to see them that much because it's mostly just talk on the phone because they are not really positive but, yeah, so I just learned not to talk to them on the phone; so that's a big step"<br><br>o "I use Skype all the time. My mother-in-law lives in (*another state*) so she sees the daughters on Skype. And I will Skype with someone. I even use Skype on my phone but-to like hold it up to my ear, I don't know. Just to visually connect with someone or I might text but I don't know what the difference is between seeing the person and just hearing their voice for me. But it's pretty rare. I almost never answer my phone."
**Providing Mutual Support**<br><br>o "And then my human support network, my Facebook people that I have. I have a group at school. We are trying to get our own place to meet so that the veterans can stay together and kind of help each other with the school issues; like a peer to peer network there."<br><br>o "…my support system is mostly with (*women from a veterans group)*. Our group is-I text them because a lot of us don't like to talk on the phone because sometimes you get too emotional. I just don't feel like talking to people, but I text a lot of my woman vet and we support each other."<br><br>o "Well, because I just-it's called 'Honor the Path of the Warrior' and I went to my first woman veteran's retreat so we set up a group on Facebook. It's a closed group. It's only for the women veteran warrior that went to the retreat so we put very confidential and we keep in the group"<br><br>o "I'm involved with the vet's center pretty heavily in (*the city*). I'm there once a week with a group and we all-all of us interact with each other like we're doing right now, be it cell phone or a text or whatever away from the VA kind of, like what are you doing in terms of this, like in terms of mental health, in terms of losing weight. And I am also involved in a group through another added facet of the VA where we are all PTSD guys and we interact with them. So it's all about networking with ourselves. Everybody in this room could be networking away from the VA. I don't know how healthy that is but it's all via communication with our cell phones, primarily."
**Obtaining Support to Cope with Symptoms or a Crisis**<br><br>o "And having that tangible connection with someone that actually someone that knew me and could do something for me; and when I got to my point where I was really at my lowest, you know, I called her in the middle of the night and (*my therapis)* arranged for someone to come pick me up....If I hadn't had that, you know, I probably wouldn't have gone to emergency room or called 9-1-1 or called one of those crisis lines where you have to talk to some stranger and you're at the bottom of your pit and you have to explain it to someone."

- “Because I have one psychologist in (*another city*) when my PTS was worse, I do not like to see anybody. I just-and then she send me an email, a poem, you know, why I should just don’t give up because I was suicidal. And she sent me that poem I just-you know, and I-just reading that poem kind of saved my life.”

Deterring Social Support

- “I think sometimes I feel safe on the computer or Facebook but…sometimes it doesn’t really get you out to meet people so that’s why I go on trying to find some social activity to do but I'm doing too much on the computer and I need to push myself out there. Facebook is good but, sometimes, too much is not good.”

- “I had my doctor ask me once, I said, ‘Well I talk to this person and that person’ and they were like, ‘Well, did you talk to them or did you text message them or did you read their posts; what exactly?’ ‘Well, I read it on the Internet and then I made a note about it.’ They were like, ‘…You need to physically talk to someone’ because I…isolate from people around me.”

Condition Management

Using Online Tools to Manage Symptoms

- “The (*smartphone*) is glued to my hand all the time and as soon as they got that PTSD app out…I loaded all my little pictures in there and my phone numbers and you can like send a text when you freak out and it will make a phone call for you.”
- “I … use the PTSD app for my blood pressure because some of the imagery and the progressive relaxation helps me lower my blood pressure.”
- “I used to do paper and pen journals but...there was an instance where someone picked one up off the bookshelf and so I don’t paper and pen journal any more. I use an online journal and I actually don’t save it on my phone. I have-there’s that eBlogger and you can have a blog that you don’t publish. So I know it’s scary that it’s floating out there somewhere but the random person who, if it were ever to surface somewhere somehow is way better than anybody I know ever reading it, so that helps me”

- “You could buy a Smartphone preloaded with apps that might help with certain conditions or…like I know they have that one packet—the app that maybe could package a bunch of apps together.”

Providing a Sense of Safety and Security

- “…when I come to the VA I get really anxious and I see things that trigger me; men that get me angry and people in uniform. I just-I'm always holding my phone because having the Smartphone…Facebook and games, it has helped [my] mental health a lot…when I come to the VA I get really anxious and I see things that trigger me—men that get me angry and people in uniform…I'm always holding my phone…”
- “(*Using my phone is)* kind of like my medication but is it healthy-I don’t know if it’s healthy, you know.”
- “You know, holding a…smartphone or whatever, you know, like I just have a rock in my pocket or something that will calm me down or focusing on something in the room to like kind of calm my anxiety. Those are just some of the things that I use.”
- “Because before I wasn’t allowed to drive because my PTSD…at least doctor won't let me drive but now I'm trying to be independent and brave and, you know, navigation helps a lot. So sometimes I get anxiety but-because I don’t know how to read a map; I need a map tell me turn left, turn right, and they need to be specific. That helps a lot, navigation.”

Signaling Reminders

- “Oh, yeah, and I forgot, now that having a smartphone I have a task list. I put it on task so when I have an appointment or, you know, I put stuff on: tomorrow, don’t forget to go to MyHealth*e*Vet or reorder”
- “I utilize Google mails appointment reminders and I set them all and so they email me and they can message on my phone to tell me to order my medication, to take my medication, to make an appointment, to go to an appointment, and then tell like things I have to check off. I'm supposed to make a few minutes for myself each day. I have this other little app that’s free that’s ‘Product TV’ or something-‘Productive TV’. It’s a little checklist thing. You can put all your little checklists in there and it reminds you when it’s done and make recurring events and that’s the only way to keep track of school and bills”
- “The telephone system for renewing medications works great, you know at least in (*town*) where I go. And like you were saying, if your prescription is expired, they will automatically send a note to your doctor to request a new one, and that happens quickly. I mean it could take three or four days to get a prescription refilled if your prescription is expired and then getting another refill is probably maybe four days. So it’s really fast. So I don’t have any complaints…”
- “And MyHealth*e*Vet is good, too, because it reminds me to, you know, when I see my-to order my medication.”

Access to and Communication with Providers

Facilitating Accurate Report of Pressing or Sensitive Issues

- “…when I was on active duty my psychiatrist and psychologist used email and it was good for *them* when I would send them an email, I suppose, having like having a difficult time and I could express how I felt at that time; for them to gauge my overall health status and not just what I say when I'm sitting in their chair. And they kept those as records to feed into my medical record so it helped them as much as it helped me.”
- “… it’s emotionally taxing to have to talk about any of it and when you have to continually explain yourself or-I think-I've stopped going to the therapy at the Vet Center maybe because I'm at my third social worker and there’s no point, like if you just have to dig it up every day, you know. I would just rather just put it all in the box and leave it on the shelf in the closet and not think about it-which is no good for me or my family but it’s way too emotionally draining when you have to repeatedly go through everything. If they had like a little note when your name pops up and say FYI, this person has this, this and this issue.”
- “It would be nice if…we have an emergency, whatever we can get on our cell phones, sent into… MyHealtheVet email so that at least our primary becomes aware….” \
- “Like sometimes I just want to either send her an email or text but sometimes I get like-she might be a challenge to convince or stuff so just send her-you know, because sometimes-like this morning I had one on one with, you know, my one on one psychologist and I would avoid talking to her and then, you know, I'm learning to let her talk and [indiscernible] so sometimes I wish I could either text or email her on some certain issue, you know, because sometimes over the phone we sometimes debate a little, you know.”
- “They could make their visual check because there would be a lot of information on how you appear and they could probably learn more about you if they saw you in your home environment and not how you shower and put on clean clothes to come to the VA.”

Promoting Timely Communication Between Veterans and their Providers

- “I use (*email*) with my (*specialist*) as well as the primary care and I think pretty much all of my people use it so it’s really handy for me and a lot of times it’s a lot faster than doing a phone call, you know. They get it

faster."

- "Yeah, I think (using email, secure messaging or texting is) faster for all of us, you know. It kind of frees up their time and they can answer it when they can. And sometimes the (*phone*) conversation goes a little bit longer because you don't always think about what you are going to say and it kind of drags on more than what it needs to."

- "About the email, the one thing that I really like is that the doctor has always got somebody waiting for him, so the nurses are the ones that were logging in to the email and doing the routing of the-and letting him know what's going on, what the-and I really like that! That's the sort of addresses the issue that you brought up."

Increasing Service Access for Disabled Veterans

- "And I worked out a plan with my psychiatrist and psychologist and we made like this email thing so like if I didn't think I could make my appointment and I couldn't get up, then I could email her and do it email back and forth or chat on MSN chat or-to let you put it on speaker phone but as long as I checked in with her if I had something. And so that worked..."

- "The first time it was-I mean I've been-in the government we used to do teleconferencing so I was familiar with that, but as far as with an in-care service of the VA system, that was the first time. But because wife was hesitant to go to see an actual doctor's office, we decided to do the teleconferencing. It was okay."

- "I would love to do therapy via Skype…I could do it in the comfort of my own home and not have to walk out the door in whatever state I'm leaving the building at and walk in front of all those men out to my car and then have to sit in my car for an hour and a half because I'm not emotionally equipped to drive home."

- "For me like sometimes my migraines are really bad that as long [indiscernible] for me and my migraines is driving so Skyping would be very-would be easier because then it wouldn't be one of my triggers so it would be easier to do that."

- "When I was on active duty I had the problem like you did. I didn't want to go out of the house. I couldn't get out of bed. I couldn't take a shower. I couldn't do anything. And they expect you, because I was on medical-waiting for my medical board, and they knew you couldn't go to work but how can they expect you to get up and drive 45 miles for a doctor's appointment when you can't even like feed yourself or take a shower?"

- "Your anxiety level is really high by the time you get there and sometimes your anxiety event is heightened by the time you have to-by the time you either get there or by the time you are leaving."

- "So when you have all those things, then it's not making an hour out of your day for an appointment. You have to budget in well, it's going to take me 15 or 20 minutes in the bathroom to clean myself up; it's going to take me an hour, hour and a half before I can drive. Now we are talking, you know, two, three hours out of my day. So when I wake up in the morning do I really want to go? Do I really want to deal with it? I don't want to deal with it. I have other stuff that's more important. I'm just not going to, whereas, if it was just a Skype phone call then, I would be more likely to participate."

- "I don't like driving in the rain. I live really far or if I'm sick, you know, because back then sometimes-in the future they could have psychotherapy at home.

Information Access

Increasing Access to Trustworthy Health Information

- “I like to go to the library a lot and I look at-I read books from there as well. The only problem is sometimes they are, you know, already past-they’re not up to date, you know, with some of the latest research… I think it’s a lot more current with some of the latest things that are going on.”
- “When the psychiatrist said I was going to need some medication I said, “Well wait a minute. I don’t want to be juiced up on drugs” and she said, “Oh, no, no, we’ve got various levels” and I said, “Well what are you going to give me?” and then I went home and went on the computer and I went searching all the medications and then looked at the other stuff I had been taking for other issues, you know is there any potential interaction? A great wealth of information on the Internet, if you just know how to go look for it”
- “I use the computer a lot and the research-I use the Mayo Clinic and other websites, the VA website. And so when the doctor tells me something then I can go and I can look and find resources or more information.”
- MyHealth*e*Vet…was a good program in order to find information and…be an advocate for my own health. There was a lot of information that I could use…and I could do it from home.”
- “So I Google it, you know, because for my Hepatitis B, like SGOT; I Google it to find out what kind of test that means, you know. It does help, you know because I read it so I know. Yeah, I just don’t go on the computer to read about PTSD now that I have the medical issue or, you know. And I'm trying to find other like natural way. I've been lately trying to find natural herb or something so I have been going on the Internet trying to find-to try to for my other health issues to take [indiscernible] affect my psychiatric meds. So, yeah, I do it, too, on different health issues.”

Obtaining Information from Peers

- “Because there’s a great blog for PTSD that covers PTSD individual unemployability, so I’m all over that. I don’t know who actually sponsors that blog but, every day there’s probably about 30 or 40 new questions or statements or something so that’s been really helpful.”
- “Sometimes I just Google search a lot. I look for different woman veteran organizations, you know. Just to research and find stuff.

Identifying Opportunities for Improved Means of Obtaining Health Information

- “I went to the National Council for PTSD and got information. That led me off into a bunch of different directions so when you do your search on the Internet (*Web)* you can either hit a good spot or a bunch of bad ones…Either way you’re getting a data overload.”
- “I use the Mayo Clinic and other websites, the VA website…The problem that I have is when I have multiple practitioners with different ideas about conditions as far as how to care for them or solve them or even their own interpretation of what the condition is—especially with PTSD.”
- “An app that would help show…some quick advice for things like PTSD for each of different conditions that you could go to that would help with those things.”
- “Log in and you could learn about all these different conditions…consolidated in one place and then it tracks what you studied and what you have learned and it gives you little reward points…making yourself feel good, but it would show your mental health provider that you are participating in your health care, you are making an effort. And then the VA could keep track a little bit better what people are using and aren’t using.”

Coordination of Care

Improving Care Coordination across Providers and Facilities

- "I ran into a situation where I was on vacation and I realized that I was running out of the medication and it would take a certain amount of time once I got back to get it refilled. So I got on and I sent my primary doctor an email saying, "Hey, I need this renewed" and I went in to look and see, no it hadn't. So I had two days later come home, went in to see the pharmacist and she gave me a week's worth of pills and she immediately put in a message to him, and that day he renewed the prescription. So it was really working well!"
- "So when I was starting to run out of medication, I went in and tried to look for my psychiatrist and I couldn't. So I sent an email to my primary letting him know what was going on and what had happened. Well, my psychiatrist had forgotten to reissue my medication and *they* were able to communicate but she and I cannot communicate through the…but I was happy that it was acted on within a day or two. Good communication there…"
- "I have a Med Alert bracelet…which I happen to have on, but you can go in and update your information on all the medications you're taking. You could be anywhere in the world and if they see that bracelet on you, they see that bracelet on you then they can get to your record and know what not to do."

Identifying Opportunities for Improved Care Coordination

- "I had an experience. My son got out of the Marine Corps about three years ago. He … has PTSD in addition to some other issues. He takes an anti-anxiety medication and so he was up here over Christmas and had forgotten his medication. I took him down … to the VA here. It wasn't that simple. I mean they could not look up his-even though he's down in (*another*) county-he goes to the (*VA*) clinic down there, they could not look up his stuff in (*his hometown VA*), and be able to access it up here. So what I had to do is we had to sit there for two and a half hours and get him signed up in (*my medical center*)... it got him through it, but it was a pain … you know?"
- "We had (*a private managed care organization*) and my wife has had some medication to take care of. And we were up in (*town A*) or (*town B*) somewhere and she didn't have any medication. And so we went to the nearest-(*private managed care organization*), and same thing. They couldn't access. There was no communication between the regions so she had to be signed up, and she was given a week's worth of medication to tide her until we got back home."
